# Efficient lossless compression of nanopore sequencing signals

**DOI:** 10.1093/bioadv/vbag157

**Published:** 2026-06-13

**Authors:** Rafael Castelli, Tomás González, Rodrigo Torrado, Álvaro Martín, Guillermo Dufort y Álvarez

**Affiliations:** Instituto de Computación, Facultad de Ingeniería, Universidad de la República, Montevideo 11300, Uruguay; Instituto de Computación, Facultad de Ingeniería, Universidad de la República, Montevideo 11300, Uruguay; Instituto de Computación, Facultad de Ingeniería, Universidad de la República, Montevideo 11300, Uruguay; Instituto de Computación, Facultad de Ingeniería, Universidad de la República, Montevideo 11300, Uruguay; Departamento de Genómica, Instituto de Investigaciones Biológicas Clemente Estable, Montevideo 11600, Uruguay; Instituto de Computación, Facultad de Ingeniería, Universidad de la República, Montevideo 11300, Uruguay

## Abstract

**Motivation:**

Efficient data compression is crucial for reducing storage and transmission costs associated to vast volumes of nanopore raw sequencing data. Surpassing the state-of-the-art compression performance has been challenging, and all recent progress in this direction either incur a computational performance over-cost or resort to lossy compression schemes, which are not always desirable.

**Results:**

In this article, we present PDZ, a lossless compression algorithm that outperforms VBZ, the current defacto standard, both in compression performance and computational efficiency. In our experimental evaluation, the compression ratio improvement ranges from 0.87% to 2.84% depending on the dataset, the compression speed is 1.09× to 2.25× faster depending on the hardware, and the decompression speed is 1.01× to 1.52× faster depending on the hardware. Compared to EX-ZD, a compression algorithm with similar compression performance, the speedup factor for both compression and decompression goes from approximately 1.39× to 1.83×, depending on the hardware.

**Availability and implementation:**

PDZ is implemented in C++ as a new compression method within the POD5 format. The source code is available as a fork of the open-source NanoporeTech library at https://github.com/Rafael-Cast/Piecewise-Differential-Zstd-Coder-POD5-Demo.

## 1 Introduction

**Figure 1:**
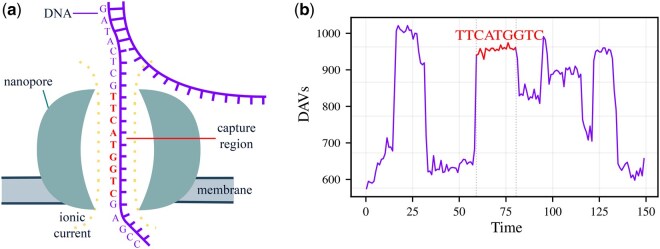
(a) A representation of a DNA molecule passing through a nanopore and perturbing the ionic current. (b) An example of a fragment of a nanopore raw signal.

Nanopore sequencers read DNA molecules by measuring the perturbation produced in an electric current flow, as a DNA strand passes through a nanometre-sized channel in a membrane (see Fig. 1a; for ease of exposition we describe DNA sequencing; RNA sequencing is analogous). This perturbation depends on the specific subsequence of the DNA strand that is occupying the *capture region* of the nanopore at certain moment, which consists of a few DNA bases. Since different DNA subsequences produce different typical current perturbations, a *basecaller* can estimate the whole DNA sequence composition from a sequence of electric current samples taken during the time the DNA strands goes through the pore in the membrane. Thus, a basecalling algorithm for nanopore sequencing takes a collection of electric current measures, referred to as *raw nanopore sequencing signals*, and produce files in FASTQ or SAM/BAM format with the DNA reads estimated from these signals.

These signals are typically sampled at 4–5 kHz, using 11–13 bits per sample, resulting in raw data volumes that are commonly very large, conveying high storage and transmission costs during the sequencing process. Moreover, since basecalling and other signal-level analysis algorithms are continuously evolving (Simpson *et al.* 2017, Wick *et al.* 2019, Ferguson *et al.* 2022, Hendra *et al.* 2022, Kovaka *et al.* 2025), raw signals are often stored for future reprocessing, making the impact of these high costs even larger. Since the incidence of lossy compression of raw nanopore sequencing data on future signal processing tools is difficult to estimate a priori, raw signals are generally encoded losslessly.

The early formats for the storage of raw nanopore sequencing signal were based on general purpose compression algorithms (Gigante 2017, Gamaarachchi *et al.* 2022). Modern formats, namely SLOW5 (Gamaarachchi *et al.* 2022) and POD5 from Oxford Nanopore Technologies (ONT) (https://github.com/nanoporetech/pod5-file-format), make use of ONT’s *VBZ* compression algorithm by default, which combines the *StreamVByte* integer encoding (Lemire *et al.* 2018) with the LZ77-based (Ziv and Lempel 1977) ZSTD compressor (https://github.com/facebook/zstd). Therefore, VBZ is currently ubiquitous in modern nanopore sequencing raw data storage and transmission applications.

VBZ, which we describe in detail in Section 2, has two key features that explain its wide adoption:

- A good compression performance (better than general-purpose compressors). (Throughout the article, *compression performance* refers to compression ratio performance; we explicitly make clear when the term performance refers to computational performance.)
- A simple design that allows efficient implementations using *Single Instruction—Multiple Data (SIMD)* CPU operations.

Indeed, both features are crucial for efficient raw data storage; in the POD5 format, compressed signals account for approximately 99.9% of the total file size, VBZ compression accounts for about 84.0% of POD5 writing time, and VBZ decompression accounts for about 98.3% of the reading time (see Sections 2.3 and 2.4 in Supplementary Material, available as supplementary data at *Bioinformatics Advances* online).

Other algorithms, like those by (Castelli *et al.* 2024) and EX-ZD from (Jayasooriya *et al.* 2025), yield better compression performance, with improvements in compression ratio of around 2.5%. However, this better compression comes at the cost of a degradation in computational efficiency in all cases, which, in practice, generally tips the balance in favor of VBZ. Other algorithms resort to lossy compression (Chandak *et al.* 2021, Jayasooriya *et al.* 2025), which, as mentioned, is not always desirable. Then the question naturally arises: is it possible to obtain a lossless compression performance improvement without sacrificing computational efficiency?

We answer this question in the affirmative. In Section 3, we introduce a novel compression algorithm, called *PDZ*, which outperforms the VBZ compression performance in all ten tested datasets and is computationally more efficient. These datasets include DNA and RNA sequencing, from various species, and using various sequencing technologies. The compression performance improvement ranges from 0.87% to 2.84% depending on the dataset. We evaluated the compression and decompression speed in eight different hardware configurations, including servers, laptops, and desktop workstations, with different operating system, CPU, and RAM configurations. Our experiments show a compression speedup factor relative to VBZ that ranges from 1.09× to 2.25×, depending on the executing hardware, and a decompression speedup factor that goes from 1.01× to 1.52×. Compared to EX-ZD, the compression performance of PDZ is similar: the relative difference is less than 0.1% for most datasets and the largest relative difference in favor of EX-ZD is 0.366%. On the other hand, the computational performance of PDZ is clearly superior: the compression speedup factor of PDZ compared to EX-ZD ranges from 1.4× to 1.83×, and the decompression speedup factor goes from 1.39× to 1.85×. These results are reported in Section 4.

We implemented PDZ using SIMD instructions in C++ as a new compression method within the POD5 format to facilitate its adoption and integration into existing nanopore analysis pipelines. The implementation was developed as a fork of the open-source NanoporeTech library, and the source code is available online (https://github.com/Rafael-Cast/Piecewise-Differential-Zstd-Coder-POD5-Demo). Additional repositories for experiments reproduction are also available (see Supplementary Material, available as supplementary data at *Bioinformatics Advances* online).

## 2 Raw nanopore sequencing signal compression

### 2.1 Nanopore sequencing

In nanopore sequencing, DNA is read by driving DNA strands through nanopores in a membrane (Fig. 1a). In its way through the pore, a DNA strand perturbs a ionic current, which is sampled and quantized over time, giving rise to a sequence of integers, $x=x_{1},x_{2},\ldots ,x_{n}$, called *data acquisition values* (DAVs). The electric current is sensitive to the specific subsequence of *k* consecutive bases (a *k*-mer) of the DNA strand that occupies the *capture region* of the pore (the value of *k* depends on the specific sequencing device). Each different k-mer generates a characteristic mean current value, which is used in a *basecalling* process to estimate the DNA strand composition from the DAV sequence x. Figure 1b illustrates a DAV sequence, highlighting in red the samples generated by the specific k-mer that lies in the capture region in Fig. 1a. The figure also illustrates the typical stepped shape of the DAV sequence, where we identify *stable regions*, corresponding to consecutive samples of a single k-mer, and *transition regions*, corresponding to times where a k-mer is being displaced from the capture region by another.

### 2.2 VBZ

**Figure 2:**
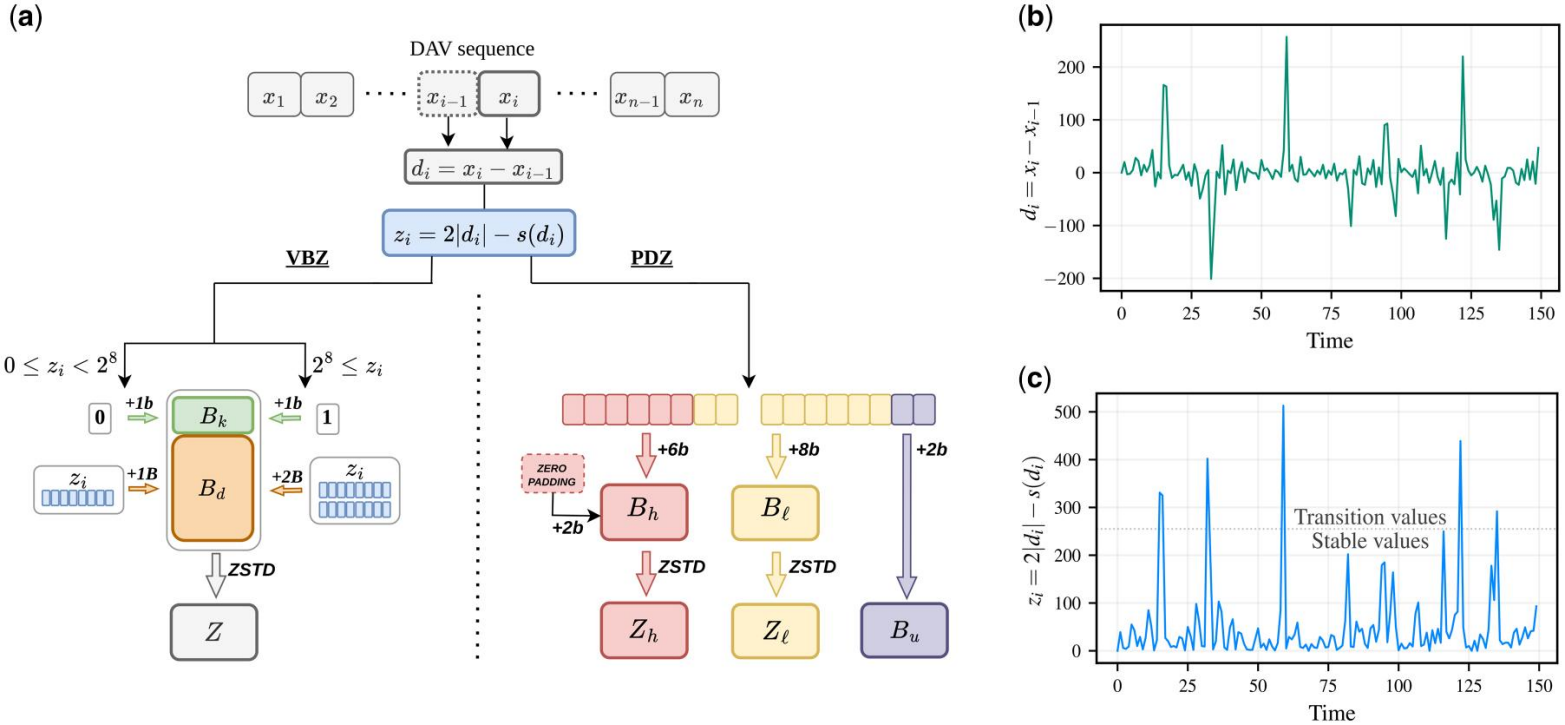
(a) Block diagram for VBZ (left) and PDZ (right). (b) Difference sequence of the raw signal of Fig. 1b. (c) Rice mapping of the difference sequence of b.

VBZ encodes x indirectly, by actually encoding a transformed sequence from which x can be readily reconstructed. The encoding consists of three main stages, which we present next. The overall compression process is illustrated on the left side of Fig. 2a.

1. **Zig-zag transform differential coding:** We define the *difference sequence* of x, denoted $d=d_{1},d_{2},\ldots ,d_{n}$, as $d_{i}=x_{i}-x_{i-1}$ for i>1, and $d_{1}=x_{1}$. The typical alternation between stable and transition regions in x translates into long runs of small-magnitude values in d (associated to stable regions), interspersed with abrupt spikes of large absolute values scattered along d (associated to transition regions). This is illustrated in Fig. 2b, and is exploited in VBZ for data compression using fewer bits to represent small absolute values, which occur frequently, and more bits to represent large absolute values, which occur more rarely. To this end, VBZ transforms d into a sequence of nonnegative integers, $z=z_{1},z_{2},\ldots ,z_{n}$, where $z_{i}$ is the Rice mapping (Rice 1991) of $d_{i}$: $z_{i}=2|d_{i}|-s(d_{i})$, with $s(d_{i})=1$ for negative $d_{i}$, and $s(d_{i})=0$ otherwise. This mapping, sometimes referred to as *zig-zag* encoding, enumerates non-negative integers in increasing order of absolute value, mapping 0,−1,1,−2,2,… into 0,1,2,3,4,…. Figure 2c shows the resulting $z_{i}$ values and illustrates how small $z_{i}$ values correspond predominantly to stable regions, whereas larger $z_{i}$ values are associated with transition regions. Since it is reversible, a decoder can obtain d from z and then reconstruct x from d.
2. **StreamVByte coding:** In this stage, VBZ builds an intermediate representation of z as follows:
  - Each value $z_{i}$ is coded as a pair $\left(k_{i},q_{i}\right)$. The *key* $k_{i}$ is a single bit equal to 0 if $z_{i}<2^{8}$, and equal to 1 otherwise. The component $q_{i}$ is the binary representation of $z_{i}$ as an unsigned integer of 8 bits if $k_{i}=0$, and 16 bits if $k_{i}=1$.
  - All keys $k_{i}$ are stored in a *key buffer*, denoted $B_{k}$, in order of occurrence.
  - The binary representations $q_{i}$ are stored in a separate *data buffer*, denoted $B_{d}$, in order of occurrence.
3. **ZSTD compression:** Finally, the concatenation of the buffers $B_{k}$ and $B_{d}$ is compressed with ZSTD.

This simple design yields a good compression performance and, at the same time, allows for SIMD parallelization both for the zig-zag transform differential coding and the StreamVByte coding.

### 2.3 Other compression algorithms

The compression performance of VBZ can be successively improved through a sequence of incrementally complex algorithm modifications (Castelli *et al.* 2024):

1. Separate ZSTD compression of buffers $B_{k}$ and $B_{d}$. This results in an average (the average is over the experimental datasets reported in (Castelli *et al.* 2024) compression ratio improvement of 0.42%.
2. Splitting $B_{d}$ into two independently-ZSTD-compressed buffers: $B_{d}^{H}$ for the 8 most significant bits of all 16-bits-long $q_{i}$, and $B_{d}^{L}$, for the 8 least significant bits of all $q_{i}$. This yields an average compression ratio improvement of 1.65% compared to VBZ.
3. Further splitting $B_{d}^{L}$ into two independently-ZSTD-compressed buffers: one for samples with $k_{i}=1$ and the other for samples with $k_{i}=0$. This results in a 1.8% compression ratio improvement over VBZ.
4. Substituting an arithmetic coder (Rissanen 1976) on an adaptive zero-order model for ZSTD in all four buffers yields a 3.66% compression ratio improvement compared to VBZ.

Compared to VBZ, some of the techniques used in variants 1–4, such as data-dependent buffer separation and adaptive models, increase data dependencies and irregular memory access patterns during execution. As a consequence, these variants are less suitable to the use of SIMD CPU instructions, which require the same operations to be applied on contiguous data elements of the same size, typically with predictable memory access patterns. In practice, this makes a full-fledged SIMD implementation of VBZ approximately twice as fast as variants 1–3, which in turn are orders of magnitude faster than variant 4 (see Castelli *et al.* 2024, for details). Similar considerations apply to other algorithms by (Castelli *et al.* 2024) and EX-ZD from (Jayasooriya *et al.* 2025).

## 3 Method

### 3.1 The algorithm

We propose a novel compression algorithm that consists of a piecewise compression of zig-zag-transformed difference samples using ZSTD, hence the name *Piecewise Differential Compressor (PDZ)*. A block diagram of the algorithm is presented in Fig. 2, side-by-side with that of VBZ for ease of comparison. PDZ consist of three main stages.

1. **Zig-zag transform differential coding:** Obtain the transformed sequence z from x as in VBZ.
2. **Piecewise split:** Split each 16-bits sample $z_{i}$ into three pieces of constant lengths h,ℓ and *u*, none of them larger than 8, where h+ℓ+u=16. Copy the *u* less significant bits of $z_{i}$ directly to an *uncompressed buffer*, $B_{u}$. Extend the next ℓ bits of $z_{i}$ to a full byte prepending 8−ℓ zeros, and add it to a buffer $B_{\ell }$. Similarly, extend the *h* most significant bits of $z_{i}$ to a full byte prepending 8−h zeros, and add it to buffer $B_{h}$.
3. **Buffer compression:** Compress the buffers $B_{\ell }$ and $B_{h}$ separately with ZSTD, obtaining compressed buffers $Z_{\ell }$ and $Z_{h}$, respectively. Encode the lengths of $Z_{\ell }$, $Z_{h}$, and $B_{u}$, and concatenate to this encoding the buffers $Z_{\ell }$, $Z_{h}$, and $B_{u}$ themselves.

The rationale behind this algorithm is to put together pieces of signal samples that exhibit similar statistics in the same buffer, separate from pieces that would better fit a different statistical model. In general, such separation accelerates data statistics learning for data compression, which yields an improvement in compression performance.

As a result of the Rice mapping, the uncompressed buffer contains the sign bit and the less significant magnitude bits of the difference sequence d. These bits are in general highly affected by noise and, thus, poorly compressible. Hence, PDZ leaves $B_{u}$ uncompressed, avoiding in this way a fruitless computational effort. The data in buffers $B_{\ell }$ and $B_{h}$, on the other hand, are in general compressible and statistically very different between each other. The most frequent samples, which correspond to stable regions of the signal, generate small values of $z_{i}$, which largely determine the data statistics in $B_{\ell }$. On the other hand, these small values of $z_{i}$ associated to stable signal regions, translate into long runs of zeros in $B_{h}$, which makes $B_{h}$ highly compressible. Zero-padding the values stored in both buffers to 8 bits align these statistical regularities with the alphabet size expected by ZSTD.

### 3.2 Parameter definition

We define values for the system parameters *u*, ℓ and *h* balancing compression performance and computational complexity considerations. With this perspective in mind, we fix ℓ=8, which allows for an efficient padding-less filling of $B_{\ell }$. Since we have h+ℓ+u=16, we are left with a single free parameter. We further constrain *u* to be a power of two, i.e. u∈{1,2,4,8}. This constraint facilitates the use of SIMD instructions, as explained in Section 3.3. From an empirical evaluation, detailed in Section 4.3, we set u=2.

### 3.3 SIMD implementation

Notice that, opposite to other compression algorithms that we have reviewed (including VBZ), the buffer separation in PDZ is independent of the sample values. As a result, $B_{\ell }$ and $B_{h}$ are exactly *n* bytes long, where *n* is the number of samples in x. The data elements of $B_{\ell }$ and $B_{h}$ are obtained through the same sequence of operations (bit masking, shifting, and buffer writing) applied to each $z_{i}$ independently. As a consequence, this sequence of instructions can be executed simultaneously on blocks of consecutive samples recurring to SIMD instructions.

Furthermore, since *u* is a power of 2, the number $\frac{8}{u}$ of samples per byte of $B_{u}$ is also a power of 2. Thus, since the number of 16-bit samples $z_{i}$ that fit into SIMD register is also a power of 2, the filling of $B_{u}$ with bits from sample-loaded SIMD registers follows a regular pattern that can be readily parallelized with SIMD instructions.

On the decoder side, the operations are symmetric to the encoder, reverting the buffer splits and sample transformations performed by the encoder. This reverse operations can also be implemented using SIMD instructions analogous to the encoder.

Our implementation of PDZ provides optimizations for both x86 and arm64 architectures, using 128-bit SSE3-class integer intrinsics and NEON instructions, respectively.

## 4 Results

To assess the performance of the proposed algorithm we executed PDZ, EX-ZD, and VBZ on ten publicly available datasets, named DS1–DS10, covering DNA and RNA sequencing, various species, and various sequencing technologies. Detailed datasets information is provided in Section 1 of the Supplementary Material, available as supplementary data at *Bioinformatics Advances* online.

In the POD5 format, the DAV sequences x are split into chunks, each compressed and stored separately. In all the experiments reported in this section we compress POD5 chunks independently, as stored in the datasets.

### 4.1 Compression performance

To measure the compression performance of an algorithm on a dataset, we compress all reads of all files of the dataset separately and calculate the *compression ratio* (*CR*) in bits per sample (bps), defined as CR=C/S, where *S* is the total number of samples in all reads of all the files in the dataset, and *C* is the total size in bits of the compressed data. Notice that smaller values of *CR* correspond to better compression performance. To compare the compression ratios of PDZ to another Algorithm A, where *A* is either VBZ or EX-ZD, we report the *percentage relative difference (PRD)*, defined as $\frac{{\text{CR}}_{2}{-\text{CR}}_{1}}{{\text{CR}}_{1}}\times 100$, where ${\text{CR}}_{1}$ and ${\text{CR}}_{2}$ are the compression ratios obtained with *A* and PDZ, respectively. Negative values indicate a better performance of PDZ. The results are reported in Table 1, where we observe that PDZ outperforms VBZ across all tested datasets, with up to 2.84% improvement in compression ratio. The compression performances of PDZ and EX-ZD are similar, with a PRD smaller than 0.1% for most datasets (8 of 10) and a maximum PRD equal to 0.366% for DS6.

**Table 1:** Compression ratio, in bits per sample (bps), for VBZ, EX-ZD, and PDZ, with PRD of PDZ with respect to each baseline.

Dataset	VBZ CR (PRD)	EX-ZD CR (PRD)	PDZ (bps)
DS1	7.143 (−2.722)	6.954 (−0.081)	6.949
DS2	7.117 (−2.841)	6.922 (−0.105)	6.914
DS3	6.887 (−2.123)	6.742 (−0.031)	6.740
DS4	6.795 (−1.882)	6.665 (0.030)	6.667
DS5	6.879 (−2.012)	6.737 (0.062)	6.741
DS6	7.074 (−0.872)	6.987 (0.366)	7.012
DS7	6.811 (−1.941)	6.675 (0.070)	6.679
DS8	6.683 (−1.676)	6.566 (0.081)	6.571
DS9	6.683 (−1.669)	6.566 (0.084)	6.571
DS10	7.346 (−1.659)	7.207 (0.233)	7.224

### 4.2 Computational efficiency

To compare the computational efficiency of the three tested algorithms, we measured the single-thread wall-clock time for compression and decompression of POD5 DAV chunks, excluding any POD5 library processing overhead (see Supplementary Material, available as supplementary data at *Bioinformatics Advances* online, Section 2.1, for methodological details). For VBZ we used the reference implementation in the POD5 library, and for EX-ZD we used the authors implementation (Jayasooriya *et al.* 2025). We compiled and executed all algorithms under identical conditions.

We conducted performance experiments on eight different systems representing diverse hardware configurations: two x86 servers (x86 S1, x86 S2), two x86 desktop workstation (x86 D1, x86 D2), two x86 laptops (x86 L1, x86 L2), and two ARM laptops (arm64 L1, arm64 L2). The technical specifications of these machines are detailed in Section 2.1.1 of the Supplementary Material, available as supplementary data at *Bioinformatics Advances* online.

Each experiment consisted of 50 independent runs, each on a different POD5 file. For each file we measured the compression and decompression time of groups of 100 chunks (except, possibly, for the last group that may be smaller), and calculated the average speed, in MB/s, along all groups of the file. Since DAV samples are 2 Bytes long, this speed is calculated as $2N2^{-20}/T$, where *N* is the number of compressed/decompressed samples and *T* is the processing time in seconds.

The average and standard deviation of the measured compression and decompression speeds on each machine are reported in Tables 2 and 3, respectively. The tables also show the speedup factors of PDZ with respect to VBZ and with respect to EX-ZD. We observe that PDZ consistently outperforms both algorithms on all machines both for compression and decompression. The compression speedup factor is larger than 1.1× in all cases, which represents an improvement of at least 10%, and in some cases PDZ is twice as fast VBZ (x86 L1 and arm64 L1). PDZ compression is at least 1.4× faster than EX-ZD in all cases. The decompression speedup factor with respect to VBZ is larger than 1.1× in most cases (except on x86 D2 and arm64 L2), and the largest factor (1.52×) represents an improvement of more the 50% in decompression time. Compared to EX-ZD, PDZ decompression is at least 1.39× faster in all cases.

**Table 2:** Compression speed (MB/s, mean ± std) for VBZ, EX-ZD, and PDZ per machine, with PDZ speedup factors relative to each baseline.

Machine	VBZ	EX-ZD	PDZ	PDZ/VBZ	PDZ/EX-ZD
x86 S1	727.8 ± 21.8	474.2 ± 11.5	806.8 ± 40.3	1.11×	1.70×
x86 S2	1030.6 ± 41.7	855.7 ± 44.8	1214.9 ± 71.9	1.18×	1.42×
x86 D1	1032.6 ± 40.7	859.2 ± 41.2	1204.5 ± 71.2	1.17×	1.40×
x86 D2	886.9 ± 35.3	569.8 ± 20.3	989.4 ± 54.8	1.12×	1.74×
x86 L1	292.0 ± 34.8	359.2 ± 24.0	656.8 ± 44.9	2.25×	1.83×
x86 L2	963.3 ± 59.4	677.0 ± 57.5	1118.1 ± 61.7	1.16×	1.65×
arm64 L1	1015.9 ± 58.7	1207.3 ± 82.9	2036.4 ± 142.0	2.00×	1.69×
arm64 L2	1214.3 ± 99.1	1048.5 ± 65.5	1749.3 ± 139.7	1.44×	1.67×

**Table 3:** Decompression speed (MB/s, mean ± std) for VBZ, EX-ZD, and PDZ per machine, with PDZ speedup factors relative to each baseline.

Machine	VBZ	EX-ZD	PDZ	PDZ/VBZ	PDZ/EX-ZD
x86 S1	1061.4 ± 50.2	648.3 ± 26.5	1164.3 ± 42.2	1.10×	1.80×
x86 S2	1363.6 ± 55.1	1143.8 ± 67.4	1589.4 ± 86.0	1.17×	1.39×
x86 D1	1343.4 ± 53.8	1132.6 ± 74.4	1576.4 ± 74.7	1.17×	1.39×
x86 D2	1254.6 ± 50.7	805.8 ± 33.1	1265.8 ± 45.8	1.01×	1.57×
x86 L1	507.3 ± 43.2	417.7 ± 22.4	770.8 ± 47.8	1.52×	1.85×
x86 L2	1262.1 ± 95.0	867.3 ± 76.9	1388.3 ± 57.4	1.10×	1.60×
arm64 L1	1247.3 ± 62.3	1014.9 ± 71.9	1625.8 ± 106.3	1.30×	1.60×
arm64 L2	1472.9 ± 76.2	935.6 ± 51.1	1522.1 ± 68.5	1.03×	1.63×

### 4.3 Parameter evaluation

**Figure 3:**
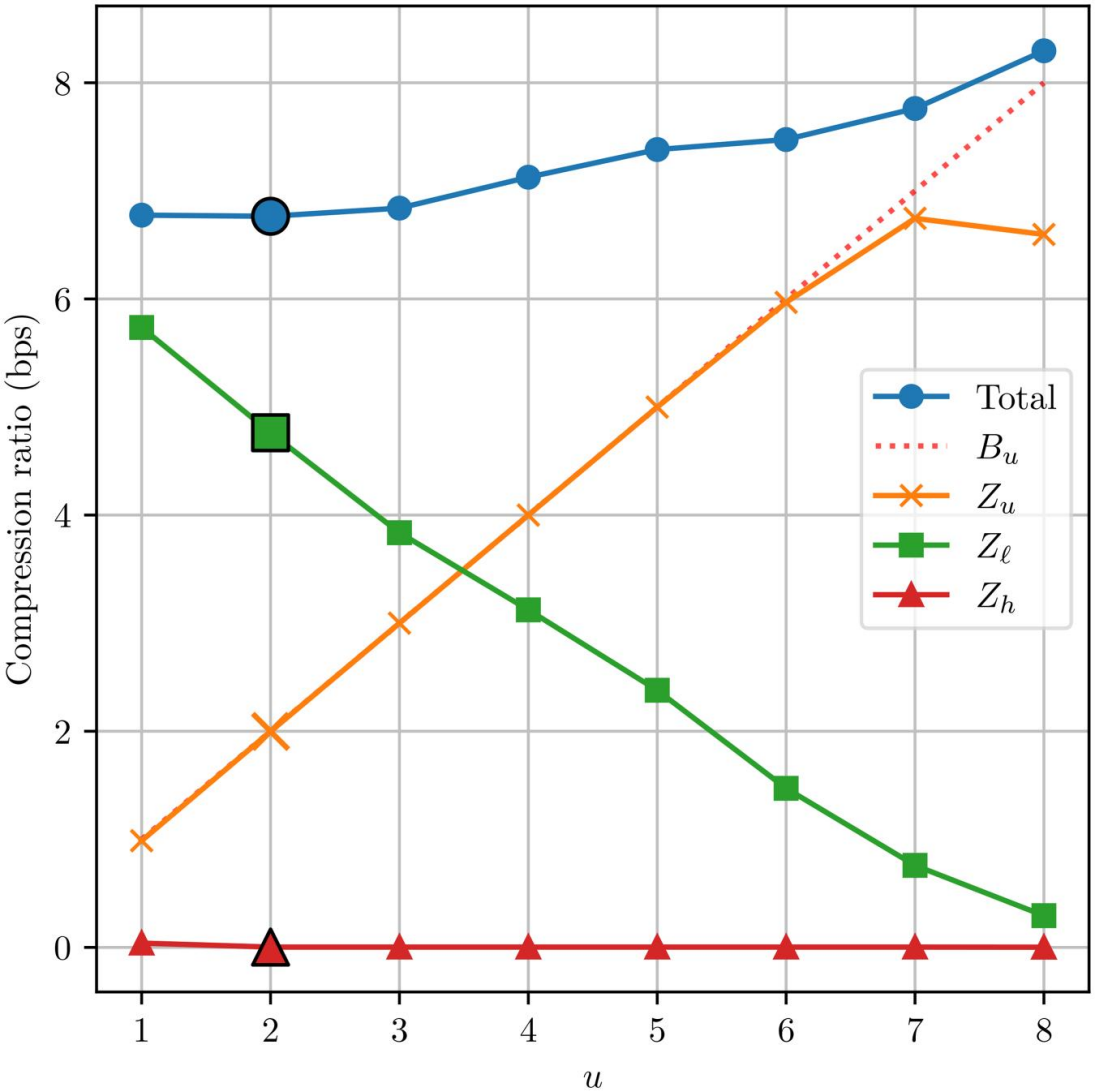
Compression performance as a function of *u*.

The definition u=2 follows from an empirical evaluation of compression performance on a fraction of the experimental data set. Figure 3 shows the CR for the buffers $Z_{h}$, $Z_{\ell }$, and $Z_{u}$ as a function of *u*, where $Z_{u}$ denotes the result of compressing $B_{u}$ with ZSTD. The figure also shows the total CR, calculated as the sum of the former three, and the CR for $B_{u}$ (uncompressed). Notice that for relatively small values of *u*, $Z_{u}$ and $B_{u}$ are essentially the same size, in line with our algorithm design that leaves $B_{u}$ uncompressed. We also observe that $Z_{h}$ has a minor impact on the total CR, which is mostly driven by the tradeoff between $Z_{\ell }$ and $B_{u}$, where the minimum CR is obtained with u=2.

## 5 Conclusions

**Figure 4:**
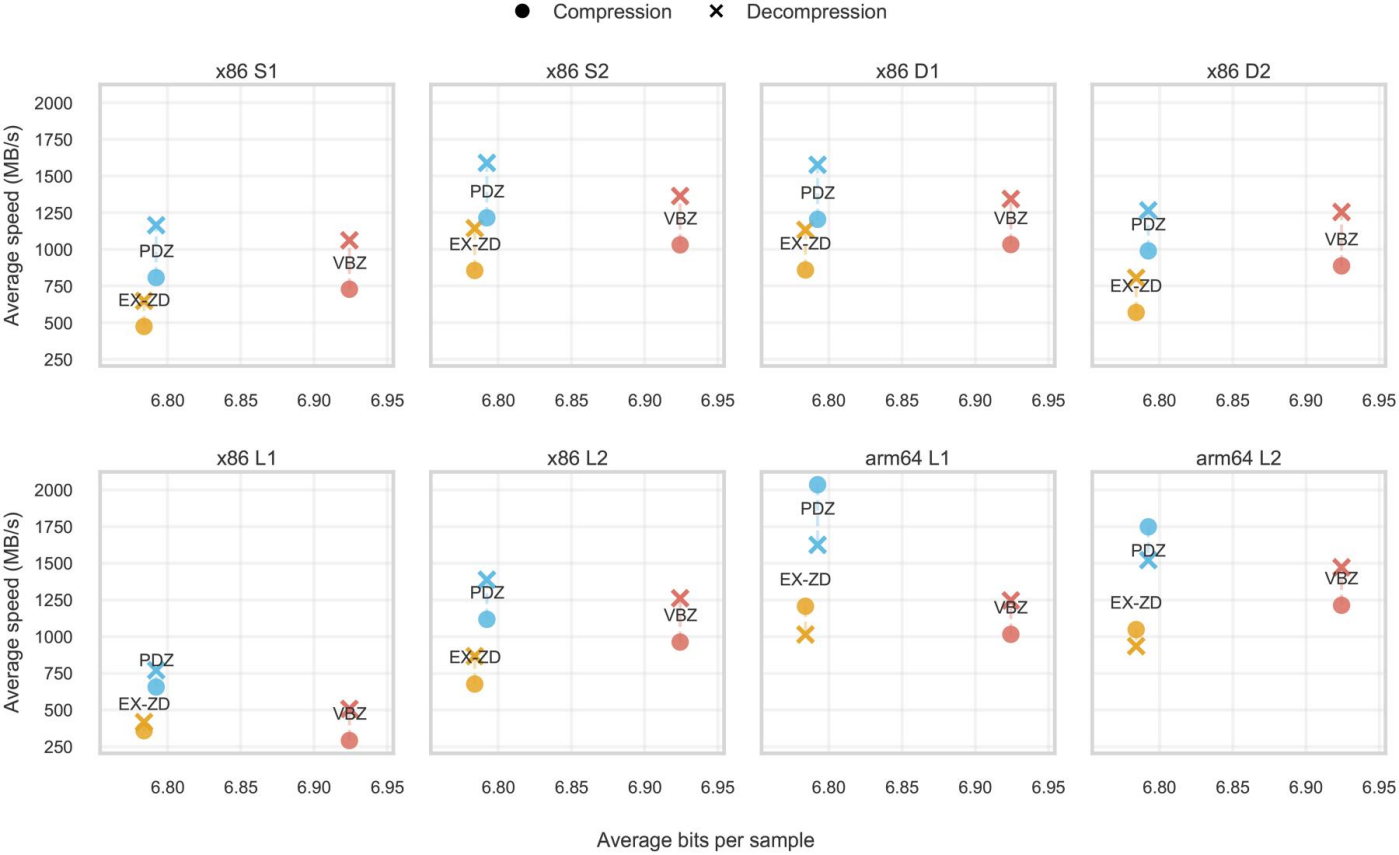
Plots of average compression ratio vs. average compression/decompression speed for PDZ, VBZ, and EX-ZD, on the eight tested execution systems. The top-left corner of each plot represents the best tradeoff between compression and speed performance.

PDZ outperforms the currently de facto standard, VBZ, both for compression performance and computational efficiency, with essentially equal memory requirements (see Section 2.5 in the Supplementary Material, available as supplementary data at *Bioinformatics Advances* online). Compared to EX-ZD, PDZ consistently surpasses the compression and decompression speeds by a factor of at least 1.39× in all tested execution platforms, with comparable compression ratios. This positions PDZ as a clear alternative for practical nanopore sequencing signal compression, as illustrated in Fig. 4. The figure shows eight plots, one for each testing hardware, of average compression ratio vs. average compression/decompression speed for PDZ, VBZ, and EX-ZD. We observe that in all cases PDZ is leaned toward the top-left corner, which represents the best tradeoff between compression and speed performance.

We have focused on lossless compression, which we regard as the most common use case. A possible direction for future work is the development and evaluation of a lossy variant following the same general algorithm design.

## Supplementary Material

vbag157_Supplementary_Data

## Data Availability

The datasets used in this study are publicly available from Oxford Nanopore Technologies open data, Zenodo, and the Sequence Read Archive. A full description of the benchmark datasets and their sources is provided in the Supplementary Material. Instructions for downloading and preparing the data are available in the GitHub repository at https://github.com/GuilleDufortFing/Nanopore-Compression-Benchmarks.
