# Supplementary material for: Efficient lossless compression of nanopore sequencing signals
Source: Bioinform Adv. 2026 Jun 13;6(1):vbag157. doi: 10.1093/bioadv/vbag157 (PMC13271238; doi:10.1093/bioadv/vbag157)
Supplement: vbag157_Supplementary_Data [file vbag157_supplementary_data.pdf]

Supplementary Material for

Efficient lossless compression of nanopore sequencing signals

Rafael Castelli[1], Tomás González[1], Rodrigo Torrado[1], Álvaro Martín[1,2], Guillermo Dufort y Álvarez[1,*]

[1] Instituto de Computación, Facultad de Ingeniería, Universidad de la República, Julio Herrera y Reissig 565, 11300 Montevideo, Uruguay
[2] Departamento de Genómica, Instituto de Investigaciones Biológicas Clemente Estable, Av. Italia 3318, 11600 Montevideo, Uruguay
[*] Corresponding author: gdufort@fing.edu.uy

All experiments reported in the main paper and in this supplementary material can be replicated using the public repository available at `https://github.com/GuilleDufortFing/Nanopore-Compression-Benchmarks`, which contains detailed instructions for reproducing each benchmark.

1 Dataset Overview

Table 1 summarizes the ten benchmark datasets used in this work. Unless stated otherwise, the reported file counts and sizes refer to the curated benchmark subsets mirrored under `data/pod5` in this repository rather than the full upstream repositories. The measurable acquisition fields in the table were extracted directly from the local POD5 files, while the short dataset descriptions follow the dataset registry used by the download workflow.

The benchmark collection spans three sequencing-technology groups: R10.4.1 DNA datasets (`DS1`, `DS3`, `DS5`, and `DS7`–`DS9`), R10.3 DNA datasets (`DS2` and `DS4`), and RNA004 direct-RNA datasets (`DS6` and `DS10`). In ONT nomenclature, the R10.x label identifies the pore and flow-cell generation used for DNA sequencing, whereas RNA004 identifies a direct-RNA chemistry family. These distinctions matter at the raw-signal level because the benchmark inputs are not acquired under one common analogue-to-digital configuration: across `DS1`–`DS10` we observe both 4 kHz and 5 kHz sampling rates, together with digitisation levels of 2048 and 8192, corresponding to 11-bit and 13-bit sample resolution. Consequently, the datasets differ not only in biological source material but also in temporal sampling density and raw dynamic range. The RNA004 datasets also represent RNA molecules rather than genomic DNA, so their signal statistics should be interpreted separately from the DNA runs.

Dataset	Species	Reference	Files	Size (GiB)	Technology	Flow cell	Kit	Rate / Bits
DS1	*D. melanogaster*	ONT open data	110	40.449	R10.4.1	FLO-MIN114	SQK-LSK114	4000 / 13
DS2	*H. sapiens* (GM24385)	ONT open data	51	38.037	R10.3	FLO-PRO111	SQK-LSK109	4000 / 11
DS3	*H. sapiens* (COLO829)	ONT open data	44	38.435	R10.4.1	FLO-PRO114M	SQK-LSK114	5000 / 11
DS4	*H. sapiens* (Cliveome)	ONT open data	20	37.400	R10.3	FLO-PRO111	SQK-LSK109	4000 / 11
DS5	*H. sapiens* (HG002)	ONT open data	34	37.967	R10.4.1	FLO-PRO114M	SQK-LSK114	5000 / 11
DS6	Synthetic (SIRV)	Zenodo 14676368	1	0.662	RNA004	FLO-MIN004RA	SQK-RNA004	4000 / 13
DS7	*L. monocytogenes*	SRR31990267	1	19.979	R10.4.1	FLO-MIN114	SQK-NBD114-24	5000 / 13
DS8	*K. pneumoniae*	SRR31990260	1	33.249	R10.4.1	FLO-MIN114	SQK-NBD114-24	5000 / 13
DS9	*K. pneumoniae*	SRR31990259	1	24.761	R10.4.1	FLO-MIN114	SQK-NBD114-24	5000 / 13
DS10	*H. sapiens* (UHR)	Zenodo 10966311	1	0.725	RNA004	FLO-PRO004RA	SQK-RNA004	4000 / 11

Table 1: Overview of the curated DS1–DS10 benchmark subsets used in this repository. File counts and sizes refer to the local benchmark subsets. The Species column gives the source organism or material: *D. melanogaster*, *H. sapiens* (sample identifier in parentheses), bacterial species, or synthetic spike-in RNA (SIRV). The Reference column links to the upstream data source: the specific ONT open data study page (EPI2ME labs), a Zenodo record, or an SRA accession. Technology, flow-cell, kit, sampling rate in Hertz, and bit resolution were extracted from POD5 run metadata; the bit resolution corresponds to $\log_2$(digitisation) when the stored digitisation is a power of two.

2 Benchmark Methodology

We use two complementary kinds of inputs. The speed and compression benchmarks operate on companion `.bin` datasets extracted from POD5 files so that the comparison focuses on signal-codec behavior with minimal container and library overhead. The size-split and time-split analyses, together with the memory benchmark, operate on native POD5 files because they quantify properties of the container itself: how much of the file is attributable to signal storage, how much of the end-to-end POD5 read or write time is spent inside VBZ compression or decompression, and how much peak memory is required during POD5 round trips between compressed and uncompressed signal layouts.

2.1 Speed Benchmark

For the computational-efficiency experiments, each selected POD5 file is converted once into a simpler binary representation that preserves the original signal chunking. The converter iterates over every POD5 read and over every signal chunk within that read. Each chunk is serialized as a 32-bit unsigned chunk length followed by the chunk samples as 16-bit signed integers. The resulting `.bin` file therefore contains the same signal chunks that would be processed from POD5, but without POD5 metadata, Arrow tables, or other container-level structures on the timed path. For the reduced-size dataset used in the article speed comparison, we took the first POD5 file from each dataset and converted its leading content with a 200 MB cutoff. The converter traverses reads in file order and applies that cutoff between reads, so each reduced input contains the leading reads and their signal chunks up to roughly 200 MB of serialized benchmark-format output.

The main reason for using `.bin` files is methodological control. The simplified format was introduced to avoid contaminating runtime measurements with behavior unrelated to the codecs under study, such as POD5 or Arrow parsing, internal library caches, implicit signal decompression performed while reading POD5, memory fragmentation caused by manipulating the full POD5 container, or other container-specific side effects that can introduce unexpected timing artifacts. Once the `.bin` files have been generated, the comparative runtime benchmarks no longer traverse POD5 or Arrow data structures. The only remaining dependency on POD5 in this benchmark family is the direct VBZ codec call used when the VBZ algorithm itself is evaluated.

Before any timing starts, the entire `.bin` file is read from disk and materialized in memory as a vector of signal chunks. Consequently, file-system I/O and disk-cache effects are excluded

from the measured interval. Timing is then performed with `std::chrono::steady_clock` from the C++ standard library, so the reported compression and decompression times are wall-clock elapsed times rather than CPU times. Chunks are processed in sets of 100: the program first compresses the complete dataset set-by-set and stores all compressed buffers, and only after the full compression pass finishes does it run the decompression pass over those buffers. For each set, it records the total number of samples, total compressed size, compression time, decompression time, and an exact correctness flag obtained by comparing the decompressed samples with the original samples.

Compression and decompression throughput are reported from the uncompressed signal size:

$$\text{throughput (MB/s)} = \frac{2 \times \text{number of samples}}{\text{elapsed time} \times 2^{20}},$$

because each raw sample occupies two bytes. The benchmark also reports

$$\text{bits per sample} = \frac{8 \times \text{compressed bytes}}{\text{number of samples}}.$$

Each algorithm and input file are benchmarked in an independent executable invocation. This process-level separation helps keep cache effects comparable across algorithms: the data are reloaded for each run, in a fresh address space, instead of timing multiple algorithms back-to-back inside the same process. In addition, because the benchmark compresses the full input before starting the decompression pass, and the evaluated inputs are intended to be much larger than the processor caches, the decompression phase is not expected to benefit materially from compression-phase cache residency of the compressed buffers.

2.1.1 Evaluation Platforms

Speed benchmarks were collected on six `x86_64` machines and two ARM machines. All machines compiled the benchmark from the same source tree using the same CMake release configuration.

x86_64 machines.

x86 server 1 Intel Core i9-10940X (3.30 GHz base, 14 cores, hyper-threading enabled, 28 logical processors); L3 cache 19.25 MiB; 256 GB RAM; Rocky Linux 8.8 (Green Obsidian), kernel 4.18.0.

x86 server 2 Intel Core Ultra 9 285K (24 cores, no hyper-threading); L3 cache 36 MiB; 256 GB DDR5; Ubuntu 24.04.4 LTS, kernel 6.17.0.

x86 desktop 1 Intel Core Ultra 7 265K (20 cores, no hyper-threading); L3 cache 30 MiB; 32 GB DDR5 at 6800 MT/s; Fedora Linux 42 (Workstation Edition), kernel 6.19.7.

x86 desktop 2 Intel Core i5-11400 (2.60 GHz base, 6 cores, hyper-threading enabled, 12 logical processors); 16 GB DDR4 at 2667 MT/s; Fedora Linux 43 (Workstation Edition), kernel 6.19.14.

x86 laptop 1 Quad-Core Intel Core i5 (1.4 GHz, 4 cores); L3 cache 6 MB; 16 GB RAM; macOS 15.7.5.

x86 laptop 2 Intel Core i7-1355U, 13th Gen (10 cores, hyper-threading enabled, 20 logical processors); L3 cache 12 MiB; 16 GB DDR4 at 3200 MT/s; Ubuntu 24.04.2 LTS, kernel 6.8.0.

ARM Neon machines. Running the benchmark on ARM required a basic port of the PDZ and VBZ codecs to the ARM Neon instruction set; EX-ZD-ZSTD already supported ARM Neon natively through its upstream StreamVByte dependency and required no changes. For PDZ, the SIMD-accelerated encoding and decoding kernels were ported by replacing the x86 SSE intrinsics with equivalent ARM Neon intrinsics. Similarly, the StreamVByte (SVB) component used inside VBZ was ported to ARM Neon equivalents. Note that VBZ uses a custom 16-bit variant of StreamVByte (SVB16) operating on `int16_t` signal samples, which is distinct from the standard upstream StreamVByte library that operates on 32-bit integers and already carries ARM Neon support; the two are not interchangeable, so the port of the SVB16 x64 SIMD path to ARM Neon was required independently. No algorithmic changes were made; only the platform-specific SIMD intrinsics were replaced so that the same data-parallel structure is preserved on both architectures.

M4 Pro laptop Apple M4 Pro (arm64, 14 cores); 24 GB unified memory; macOS 15.6.1 (Darwin kernel 24.6.0).

M4 laptop Apple M4 (arm64, 10 cores: 4 performance + 6 efficiency); 16 GB unified memory; macOS 26.4.1.

2.2 Compression Benchmark

The compression benchmark uses the same `.bin` datasets as the speed benchmark, but it measures compression effectiveness instead of runtime. The binary input is streamed chunk-by-chunk. For each chunk, the selected algorithm compresses the raw signal, immediately decompresses the result, and verifies exact equality with the original samples. The benchmark records the number of samples, compressed size in bytes, and a correctness flag for every chunk. For the compression results reported in this project, we use one curated compression run for each full `.bin` dataset `DS1` through `DS10`.

The main summary statistic is again the compression density,

$$\text{bits per sample} = \frac{8 \times \text{compressed bytes}}{\text{number of samples}}.$$

Per-file summaries aggregate the total number of chunks, total number of samples, total compressed bytes, weighted bits per sample, and the number of successful and failed round trips. Because this benchmark does not measure time, it can stream the `.bin` file sequentially without affecting the quantity of interest.

2.3 Size-Split Analysis

The size-split analysis is a POD5 file-layout analysis rather than a runtime benchmark. It reads each native POD5 file and accumulates four file-level quantities: the total file size on disk, the total uncompressed signal size, the total compressed signal size reported by the POD5 reader, and the total number of signal rows. The uncompressed signal size is computed as two bytes per sample. The compressed signal size is obtained directly from the POD5 metadata for each read. For the size-split analysis we aggregate all 51 POD5 files from `DS2`.

To interpret how much of a POD5 file is occupied by signal-related storage, the analysis also reports an estimated signal-table footprint:

$$\text{signal-table bytes} = \text{compressed signal bytes} + 4 \times \text{rows} + 16 \times \text{rows}.$$

The first term is the payload of the compressed signal itself, while the fixed per-row terms approximate the bytes needed to store signal-table bookkeeping. This expression should be understood as an estimate used for structural interpretation, not as a byte-for-byte reconstruction

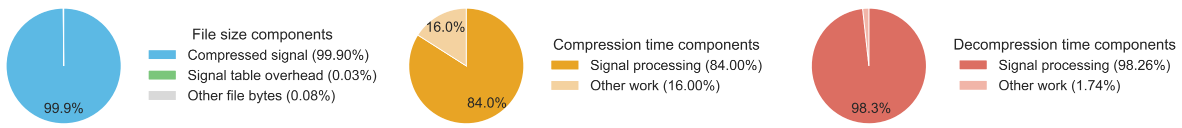

Figure 1: Workload decomposition for the curated DS2 POD5 set used in the split analyses. Left: file-size partition into compressed signal, estimated signal-table overhead, and remaining file bytes. Middle: compression-time partition into signal processing and other work. Right: decompression-time partition into signal processing and other work. Percentages are aggregated over the 51 POD5 files in the selected article run.

of the full POD5 schema. The reported percentages are the compressed signal fraction of the total POD5 size and the estimated signal-table fraction of the total POD5 size.

As shown in Figure 1 (left), compressed signals account for 99.9% of the total file size.

2.4 Time-Split Analysis

The time-split analysis measures how much of the end-to-end POD5 processing time is spent specifically inside VBZ compression or decompression, as opposed to the rest of the POD5 container handling. Unlike the previous two benchmarks, this analysis operates on native POD5 files. For the split results reported in this project, we use the curated DS2 time-split run, which covers the same 51 POD5 files as the selected size-split run. The combined workload-decomposition figure is therefore computed on this common 51-file DS2 set.

Each POD5 file is processed twice in separate runs. In the compression run, the input POD5 file is first fully materialized in memory, and the timed region then covers creation of a new POD5 file whose signal is written with VBZ compression. In the decompression run, timing starts before the input file is opened and covers the complete POD5 read and signal reconstruction path. The total elapsed time is measured in the benchmark executable with `std::chrono::steady_clock` from the C++ standard library. Independently, the codec-only VBZ time is obtained from the instrumented POD5 implementation, which also uses `std::chrono::steady_clock` and accumulates the individual internal VBZ compression or decompression intervals as `std::chrono::nanoseconds`. The decompression path also computes a checksum over the recovered data so that the compiler cannot optimize the read away.

For both compression and decompression, the benchmark reports

$$\text{codec share (\%)} = 100 \times \frac{\text{VBZ codec time}}{\text{total elapsed time}},$$

together with the number of measured codec intervals. This benchmark is therefore not a throughput comparison between alternative algorithms. Instead, it quantifies how much of native POD5 processing time is attributable to VBZ itself and how much is attributable to the surrounding POD5 machinery.

Figure 1 summarizes the combined size-split and time-split decomposition for the curated 51-file DS2 set used in this project. As mentioned, compressed signal storage accounts for almost all POD5 bytes (about 99.90%). Signal compression accounts for about 84.0% of the writing time, and signal decompression accounts for about 98.3% of the reading time. This shows that codec work dominates the read path even more strongly than the write path.

2.5 Memory Benchmark

The memory benchmark measures peak resident set size for native POD5 decompression and compression stages between compressed and uncompressed signal layouts. Unlike the speed and

compression benchmarks, it operates directly on POD5 files and therefore captures both signal-codec effects and the surrounding POD5 read/write machinery. The benchmark was run on Linux. EX-ZD-ZSTD was not evaluated in the memory benchmark because it is not integrated into the POD5 library.

The benchmark uses a standalone `copy` executable that rewrites a POD5 file with a selected signal compression mode. Given an input path, an output path, and one of `--VBZ`, `--PDZ`, or `--uncompressed`, the program opens the source POD5 file, copies the run metadata, iterates through the POD5 read batches, materializes the full signal array for each read through the POD5 C API, and writes a new POD5 file whose signal is stored with the requested compression setting. This preserves the original POD5 structure and metadata while changing only the signal-storage mode.

For each POD5 file and algorithm, the benchmark first performs an unmeasured setup conversion from the original input to the target compressed representation. It then measures two stages separately: a decompression stage from the prepared algorithm-compressed POD5 file to an uncompressed POD5 file, and a compression stage from that uncompressed POD5 file back to the selected compressed representation. The runner invokes GNU `time` via `/usr/bin/time`, which emits both wall-clock elapsed seconds and peak resident set size; in the present work, the reported outputs use only the stage-specific peak resident set size together with the corresponding input and output sizes.

The reported outputs from this benchmark are therefore the decompression peak RSS and the compression peak RSS, treated as separate measurements rather than combined into a round-trip statistic. For the memory results reported in this project, we use two curated article runs: the 51-file `DS2` run and the single-file `DS7` run for `LM41_RKI_merged.0_0.pod5`. The reported measurements therefore cover 52 native POD5 files in total, with separate compression and decompression RSS points for both VBZ and PDZ. This benchmark is also not used to estimate throughput: speed is reported by the dedicated speed benchmark. Instead, the memory benchmark quantifies the peak memory required by the native POD5 decompression and compression paths in which signal compression is changed while the rest of the container handling remains on the measured path. Figure 2 shows that peak RSS grows with file size for both codecs, and that the compression stage consistently lies above the decompression stage, while VBZ and PDZ follow closely similar scaling trends.

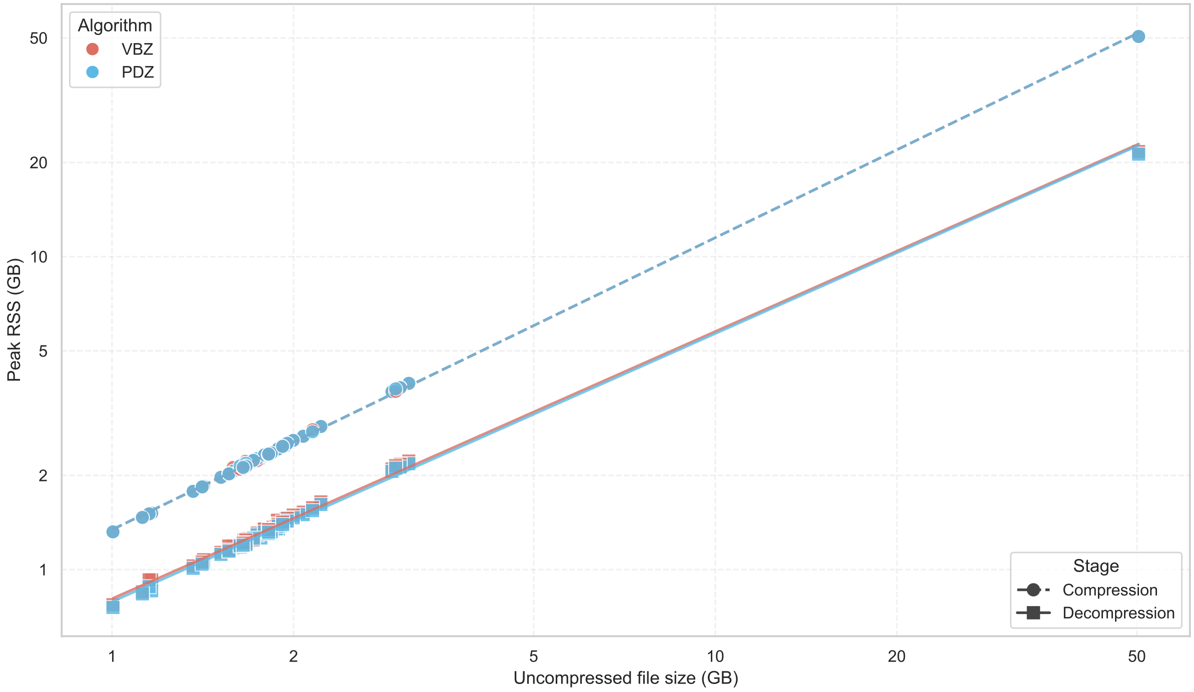

Figure 2: Per-file peak resident set size versus uncompressed file size for the curated `DS2` and `DS7` POD5 files used in the memory benchmark. Points show `VBZ` and `PDZ` measurements, with circles for compression and squares for decompression. Both axes are logarithmic. The lines are least-squares fits in log-log space, so they summarize scaling behavior with file size rather than linear growth in the original units.
